# Systemically comparing host immunity between survived and deceased COVID-19 patients

**DOI:** 10.1038/s41423-020-0483-y

**Published:** 2020-06-15

**Authors:** Feng Wang, Hongyan Hou, Yin Yao, Shiji Wu, Min Huang, Xiao Ran, Hongmin Zhou, Zheng Liu, Ziyong Sun

**Affiliations:** 1 Department of Laboratory Medicine, Tongji Hospital, Tongji Medical College, Huazhong University of Science and Technology, Wuhan, China; 2 Department of Otolaryngology-Head and Neck Surgery, Tongji Hospital, Tongji Medical College, Huazhong University of Science and Technology, Wuhan, China; 3 Department of Emergency Medicine, Tongji Hospital, Tongji Medical College, Huazhong University of Science and Technology, Wuhan, China; 4 Department of Cardiothoracic and Vascular Surgery, Tongji Hospital, Tongji Medical College, Huazhong University of Science and Technology, Wuhan, China

**Keywords:** Viral infection, Prognostic markers

Novel severe acute respiratory syndrome coronavirus 2 (SARS-CoV-2) has caused the disastrous pandemic of Coronavirus Disease-2019 (COVID-19).^1–3^ Although the epidemiological, clinical, and immunological characteristics of COVID-19 have been reported,^4–6^ the kinetics of immune responses and their association with clinical outcomes remain poorly understood. Here, we summarize the characteristics of cellular immune responses in a total of 157 COVID-19 patients enrolled in Tongji Hospital between February and March 2020 and compare the properties between 95 survived and 62 deceased patients with different onset time. The demographic and clinical characteristics of these patients are shown in Supplementary Table 1. No significant difference in age, gender, clinical symptoms, and imaging features was recorded between survived and deceased patients.

Since dysregulation of immune responses and temporal dynamics in T-cell activation have been noted in patients with COVID-19,^4,7,8^ we thus classified the patients into early (≤10 days), middle (11–20 days), late (21–30 days), and end (>30 days) stages of disease based on the time from symptom onset. The numbers of cases in the early, middle, late, and end stages were 5, 23, 33, and 34 in survived patients, and were 5, 12, 32, and 13 in deceased patients, respectively. Generally, deceased patients demonstrated higher number of neutrophils and lower number of lymphocytes compared to survived patients (Supplementary Table 2).

To gain greater insight into the kinetics of immune cells, flow cytometry was performed to explore the number, phenotype, and function of T, B, and dendritic cells (DC) as well as monocytes in survived and deceased patients (Supplementary Fig. 1). Although a comparable CD4^+^ T-cell count was noted between survived and deceased patients in the early stage, deceased patients displayed dramatically lower CD4^+^ T-cell count than survived patients in the middle and late stages, indicating the distinct immune response kinetics between two groups (Fig. 1a). We next examined the activation and effector function of CD4^+^ T cells by analyzing HLA-DR expression and IFN-γ producing ability. We found that the frequencies of HLA-DR^+^ and IFN-γ^+^ cells within CD4^+^ T cells were markedly increased in the middle stage compared to those in the early stage, which then rapidly declined in the late stage and kept a low level in the end stage in decreased patients (Fig. 1a). However, the frequencies of HLA-DR^+^ and IFN-γ^+^ cells within CD4^+^ T cells in survived patients continuously increased after symptom onset, and maintained at a certain level in the late and end stages (Fig. 1a). Thus, decreased patients demonstrated much higher frequency of HLA-DR^+^ cells within CD4^+^ T cells than survived patients in the middle stage, whereas both HLA-DR expression and IFN-γ producing ability of CD4^+^ T cells in the late stage in deceased patients were significantly lower than those in survived patients (Fig. 1a). Moreover, lower CD45RO expression on CD4^+^ T cells was noted, again, in deceased patients in comparison with that in survived patients in all stages (Fig. 1a). However, the expressions of CD45RA and CD28 on CD4^+^ T cells in the late and end stages were significantly higher in deceased patients than those in survived patients (Fig. 1a).

**Fig. 1:**
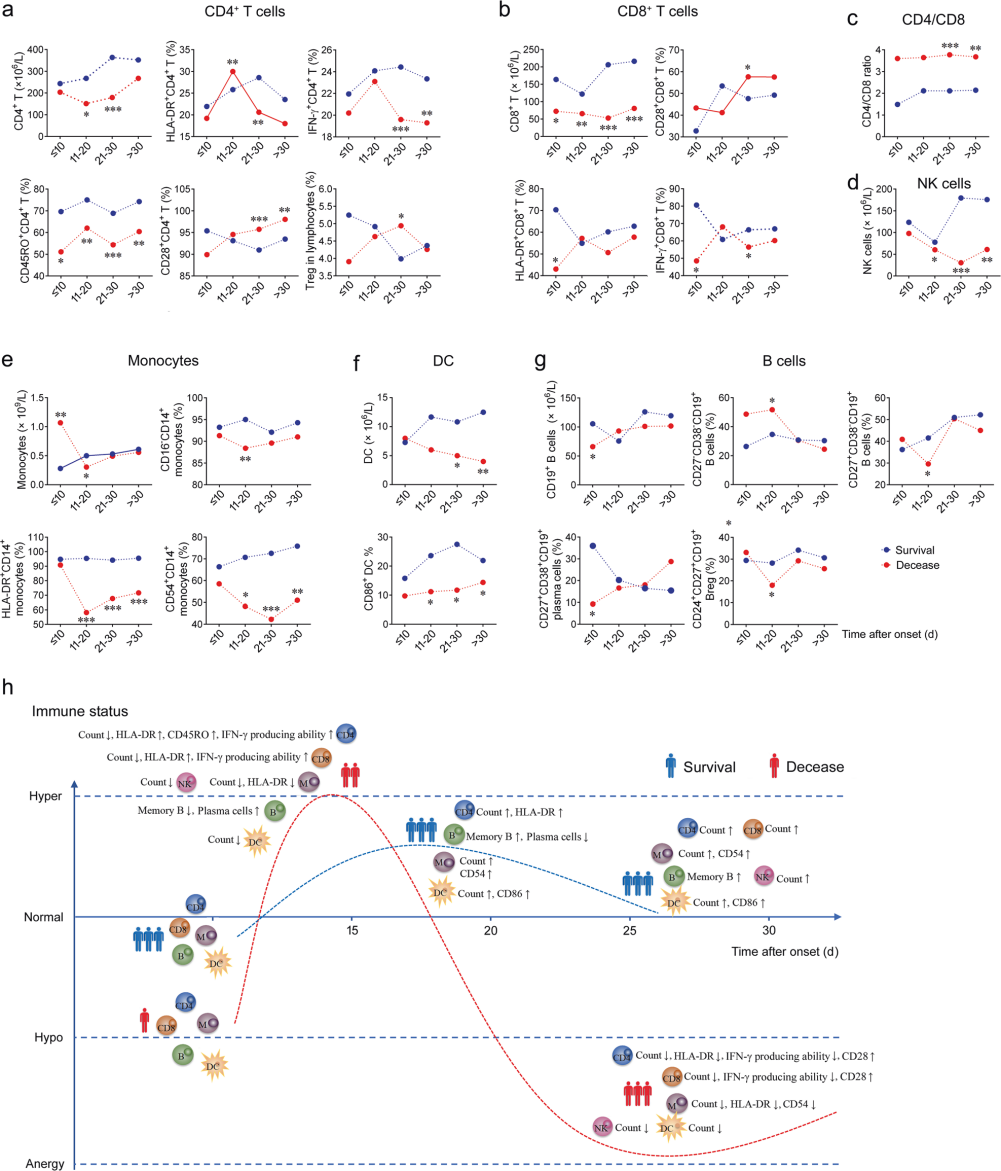
Cellular immune responses during COVID-19. **a** Absolute number of CD4^+^ T cells, frequencies of HLA-DR^+^, IFN-γ^+^, CD45RO^+^, and CD28^+^ cells within CD4^+^ T cells, and CD25^high^CD127^low^ regulatory T cells within lymphocytes in COVID-19 patients. **b** Absolute number of CD8^+^ T cells and frequencies of CD28^+^, HLA-DR^+^, and IFN-γ^+^ cells within CD8^+^ T cells in COVID-19 patients. **c**, **d** Ratio of CD4/CD8 and absolute number of NK cells (**d**) in COVID-19 patients. **e** Absolute number of monocytes and frequencies of CD16^−^, HLA-DR^+^, and CD54^+^ cells within monocytes in COVID-19 patients. **f** Absolute number of DC and frequency of CD86^+^ cells within DC in COVID-19 patients. **g** Absolute number of CD19^+^ B cells and frequencies of B-cell subsets within total B cells in COVID-19 patients. The mean values of results in different stages were shown in line graph. Data are compared between survived and deceased patients in the same stage of disease. *p < 0.05, **p < 0.01, ***p < 0.001 (Mann–Whitney *U* test). **h** The simulated diagram shows the characteristics of immune responses in survived and deceased COVID-19 patients with different onset time (↑or↓means that the mean value in the described stage is increased or decreased compared with the result in the early stage, respectively)

In all stages, the number of CD8^+^ T cells in deceased patients was obviously lower than that in survived patients (Fig. 1b). Notably, deceased patients had significantly lower frequencies of HLA-DR^+^ and IFN-γ^+^ cells within CD8^+^ T cells compared with survived patients in the early stage (Fig. 1b). Although HLA-DR expression and IFN-γ producing ability of CD8^+^ T cells also showed an increased trend from early to middle stage in deceased patients, comparable frequencies of HLA-DR^+^ and IFN-γ^+^ cells were observed in the middle stage between survived and deceased patients (Fig. 1b). Similarly, IFN-γ producing ability of CD8^+^ T cells in the late stage in deceased patients was significantly lower than that in survived patients (Fig. 1b). In addition, deceased patients displayed significantly lower CD4/CD8 ratio compared with survived patients in the late and end stages, but there was no noticeable difference in CD4/CD8 ratio among different stages in both survived and deceased patients (Fig. 1c).

In contrast to the increase of NK cell count in survived patients in the late and end stages compared with that in the early and middle stages, deceased patients showed a gradual loss of NK cells after onset of symptoms (Fig. 1d). Although deceased patients had significantly higher monocyte count than survived patients in the early stage, the monocyte count was rapidly decreased post symptom onset in deceased patients, which resulted in the comparable monocytes in the late and end stages between two groups (Fig. 1e). In addition, the frequencies of CD16^−^CD14^+^ classical, HLA-DR^+^, and CD54^+^ monocytes within monocytes showed a decreasing trend after early stage in deceased patients; thus a remarkably lower frequencies of HLA-DR^+^ and CD54^+^ monocytes were noted in deceased patients than in survived patients in middle, late, and end stages (Fig. 1e).

The number of DC gradually decreased from early to end stage of disease in deceased patients, which was significantly lower in deceased patients than that in survived patients in the late and end stages (Fig. 1f). Similarly, the frequency of CD86^+^ DC was dramatically decreased in deceased patients than that in survived patients in the middle, late, and end stages (Fig. 1f). Intriguingly, in the early stage, the frequency of CD27^+^CD38^+^ plasma cells was significantly lower in deceased patients compared with survived patients, suggesting early initial of humoral response may associate with better COVID-19 outcome (Fig. 1g). In addition, the frequencies of CD27^+^CD38^−^ memory B cells and CD24^+^CD27^+^ regulatory B cells were significantly lower in deceased patients than those in survived patients in the middle stage (Fig. 1g). Based on the above results, a schematic overview of dynamics of cellular immune responses in survived and deceased COVID-19 patients was developed (Fig. 1h). In the early stage, deceased patients displayed significant reduction of CD8^+^ T-cell count and frequency of plasma cells compared with survived patients, accompanying reduced activation and IFN-γ producing ability of CD4^+^ and/or CD8^+^ T cells. In the middle stage, although the numbers of CD4^+^ and CD8^+^ T cells further decreased, HLA-DR expression and IFN-γ producing ability of them rapidly increased in deceased patients. In the late stage, the numbers of CD4^+^ and CD8^+^ T cells, NK and DC were all remarkably decreased in deceased patients compared with survived patients; HLA-DR expression and IFN-γ producing ability of CD4^+^ and/or CD8^+^ T cells were decreased in deceased patients but with high expressions of CD28 and CD45RA. We also longitudinally evaluated the cellular immune responses in several patients from admission to discharge or death, and similar results were observed in representative two patients (Supplementary Fig. 2).

Collectively, for the first time, our data indicate that hypofunction, hyperaction, and anergy may represent the characteristics of immune responses in deceased COVID-19 patients in the early, middle, and late stage, respectively. Our study not only extends our understanding of kinetics of immune responses during COVID-19, but also may have implications for the treatment of COVID-19.

## Supplementary information

Supplementary Figure 1

Supplementary Figure 2

Supplementary Table 1

Supplementary Table 2
