# Supplementary figures and images for: Systemically comparing host immunity between survived and deceased COVID-19 patients
Source: Cell Mol Immunol. 2020 Jun 15;17(8):875–7. doi: 10.1038/s41423-020-0483-y (PMC7295144; doi:10.1038/s41423-020-0483-y)

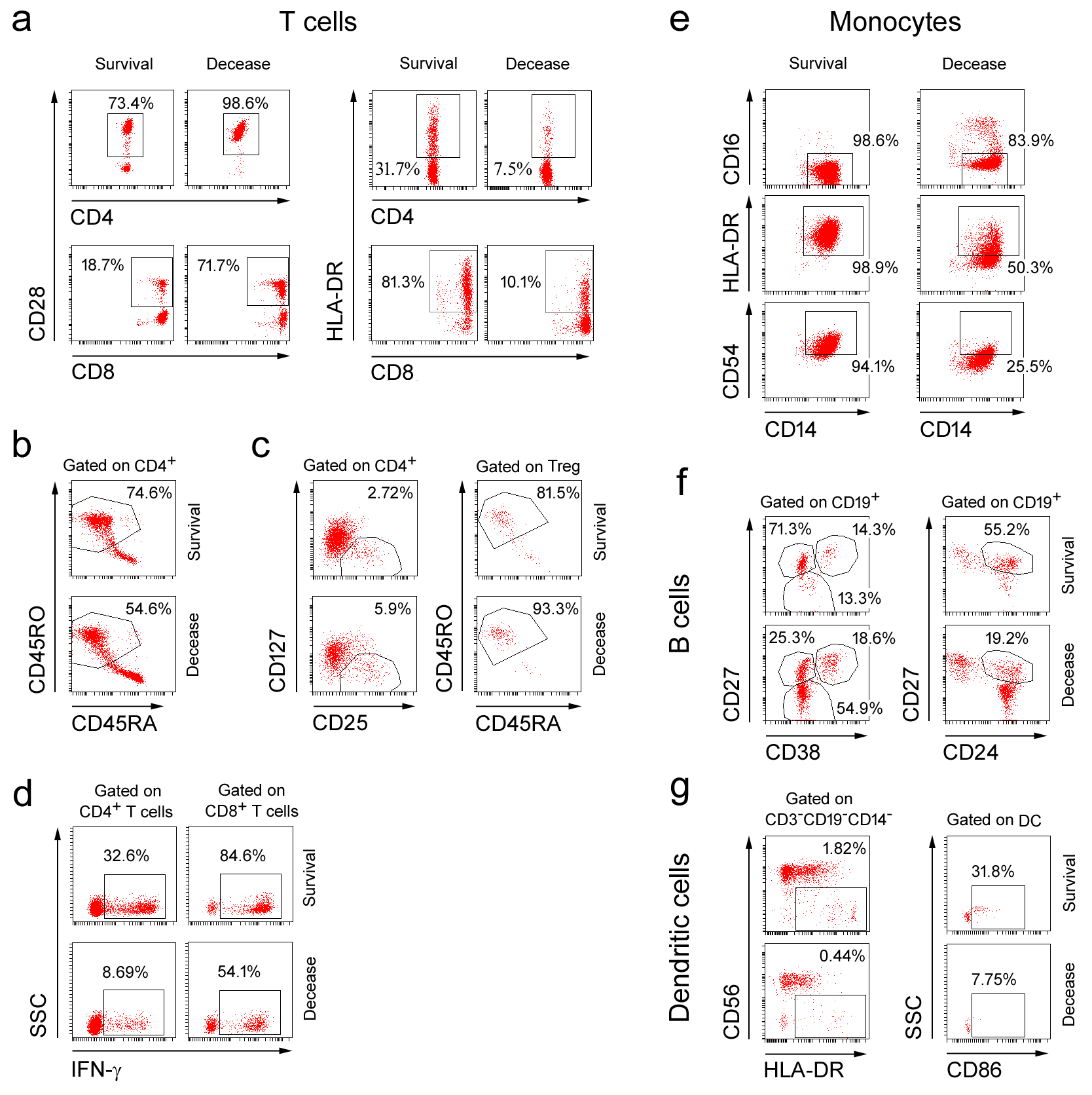

Supplement: Supplementary file 1 — Supplementary Figure 1 [file 41423_2020_483_MOESM1_ESM.tif]

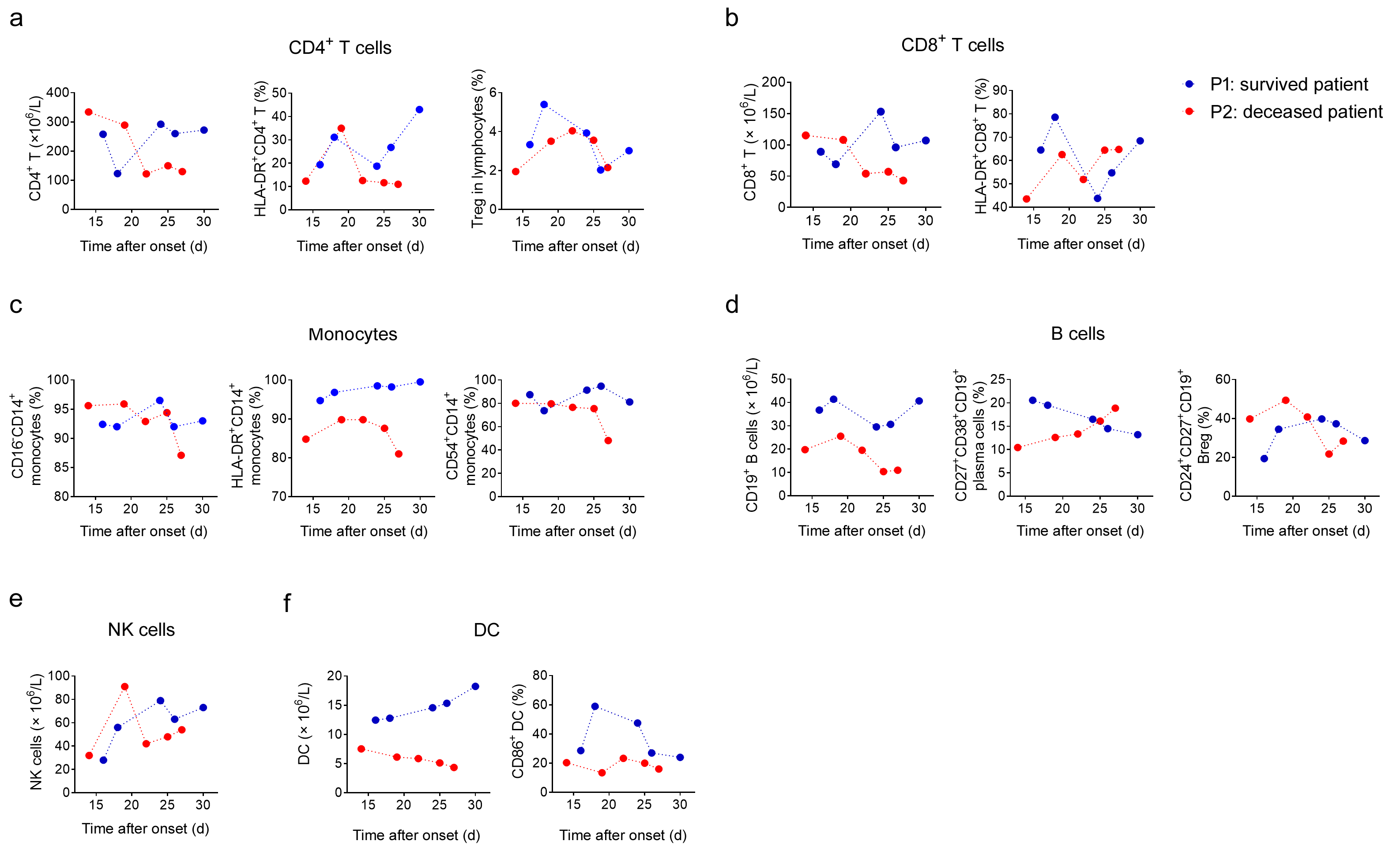

Supplement: Supplementary file 2 — Supplementary Figure 2 [file 41423_2020_483_MOESM2_ESM.tif]
